# Group coaching for career development: Supporting the endangered early career researcher

**DOI:** 10.1017/cts.2025.10089

**Published:** 2025-07-10

**Authors:** Lucy Palmer, Anupama Wadhwa, Susan Matulevicius, Anand Rohatgi, Heidi T. Jacobe

**Affiliations:** 1 Office of Clinical Research, Office of the Provost, University of Texas Southwestern Medical Center, Dallas, TX, USA; 2 Department of Anesthesiology and Pain Management, University of Texas Southwestern Medical Center, Dallas, TX, USA; 3 Outcomes Research Consortium, University of Texas, Houston, Texas, USA; 4 Faculty Wellness, Office of the Provost, University of Texas Southwestern Medical Center, Dallas, TX, USA; 5 Division of Cardiology, Department of Internal Medicine, University of Texas Southwestern Medical Center, Dallas, TX, USA; 6 Department of Dermatology, University of Texas Southwestern Medical Center, Dallas, TX, USA

**Keywords:** Coaching, workforce development, burnout, appreciative inquiry, clinical research

## Abstract

Early career researchers have unique demands, many of which contribute to increased stress, decreased professional fulfillment, and burnout. Consequently, academic institutions and government organizations, such as the National Institutes of Health, are beginning to embrace structured coaching as a tool to support physician wellbeing. To date, such coaching programs have demonstrated promising results, but little is known about whether early career research faculty find coaching feasible, accessible, or helpful. To explore this question further, we developed a novel group coaching intervention for clinician researchers and scientific faculty at the University of Texas Southwestern Medical Center based on the concept of appreciative inquiry, grounding the program in a positive and hopeful approach to the challenges faced by clinicians and researchers. Results from our program indicate this intervention is feasible, satisfactory, and helpful, with participants reporting enhanced self-reflection and empowerment. Effective for a wide array of research faculty, our program brought together diverse faculty, fostered connections, and encouraged future collaborations among this translational group. This suggests that our program provides a foundational blueprint that can be used by other academic medical centers who aim to develop group coaching efforts.

## Introduction

In the corporate environment, coaching is accepted as integral to development and has been successfully deployed in support of organizational change, goal attainment, resilience, and workforce wellbeing [1,2]. In this setting, coaching is viewed as a complement to mentoring and vital to early career development. Nevertheless, in academia, coaching is often used as a tool for remediation [3], producing stigmatization, or only available to senior leadership. In contrast, in academic medicine, mentorship of junior faculty remains the mainstay for career development [3,4], while the complementary role of coaching is not well established, particularly among early career investigators [5,6].

This is of concern because numerous publications highlight the decreasing number of clinician scientists and laboratory-based research faculty, underscoring a major threat to the biomedical workforce [8–10]. Furthermore, persistent lack of diversity in the research workforce is a persistent barrier [8,11]. These concerns prompted entities like the National Institutes of Health (NIH), among others, to create funding mechanisms to provide salary support for early career investigators and encourage programs that support their career development, that are highly focused on mentoring relationships for early career investigators [7]. Underuse of coaching in this group may stem from a lack of understanding of the differences between coaching and mentoring. Coaching focuses on the client understanding the “why” behind their behaviors and maximizing their effectiveness in a goal-driven context, whereas mentoring often provides advice or guidance through examples of personal experience in a more directive [7]. Consequently, coaching may provide unique benefits to research track faculty that complements traditional scientific and career mentoring.

Institutions are beginning to embrace coaching and group peer mentoring programs as a tool to support physician wellbeing [5,13]. Such programs have seen promising results [14–16], including a coaching program established at the University of Texas Southwestern Medical Center (UTSW) for female faculty [12], but few exist with established, adoptable infrastructures. Some coaching programs have been run in a group setting [17], promoting peer connection and support through the coaching process [18], although group coaching is less well characterized than traditional 1:1 coaching interventions. Despite these initial promising results, it is unknown if early career research track faculty find coaching acceptable, feasible, or helpful. Therefore, we co-created and implemented a novel pilot group coaching intervention at UTSW based on the concept of appreciative inquiry [19–21]. We assessed feasibility, satisfaction, and effectiveness of the coaching program through an iterative process that utilizes feedback from the participants to guide overall structure and development.

## Materials and methods

These activities were reviewed by the UTSW Human Research Protection Program and determined to be part of program evaluation. Consequently, the subject matter of this paper was deemed non-regulated research, so no institutional review board approval or oversight was required.

### Overview

To combat the declining numbers of early career clinician scientists and laboratory-based research faculty (research track faculty), we designed our group coaching program to determine satisfaction, feasibility, and effectiveness of both the coach and intervention to inform institutional deployment.

### Program design

**Figure 1.**
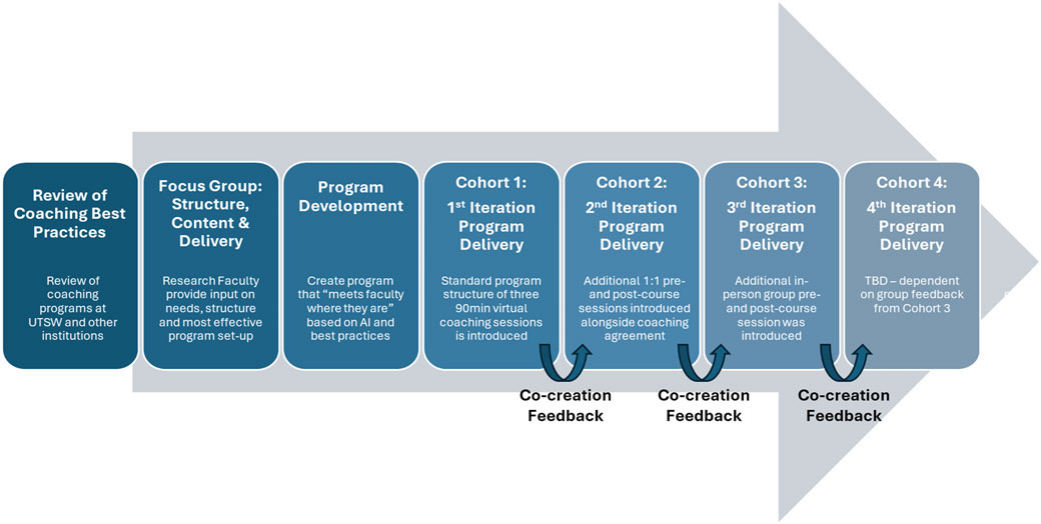
UTSW Group Coaching Program Development. This figure displays the process of UTSW Group Coaching Program Development, including the programmatic updates and changes made in response to the feedback of participants in our dynamic co-creation process (time frame: approximately 12 months, appreciative inquiry (AI)).

We completed a review of previous coaching programs at UTSW and across other institutions [5, 14–18] to determine best practices (Figure 1). Coaching has been a growing initiative at UTSW, championed by the Office of Faculty Wellness, so we utilized their team’s experience and reviewed their practices as part of our feasibility assessment [12].

A team was assembled with representatives from the Office of Faculty Wellness, workforce development faculty from the Office of Clinical Research, and certified coaches who met with a focus group of current clinician scientists to identify the most suitable programmatic content and delivery modality (Figure 1). The focus group mirrored previous findings, indicating that clinician scientists and laboratory-based researchers desire: 1) to be “met where they are” geographically for wellness programs to be successful [22]; 2) flexibility to accommodate challenging schedules; and 3) the option to select discussion topics. Ongoing programmatic refinement continued with feedback from participants in each cohort, with the aim of engaging our target audience in a program co-creation process. With continuous feedback and input from all three cohorts to date, we produced a robust and replicable group coaching program outline (Figure 1, Table 1).

**Table 1.** Outline of group coaching program at UTSW

	Group Introduction	Pre-Course 1:1	Group Session 1	Group Session 2	Group Session 3	Post-Course 1:1	Follow-up Group Check-in
Format	In-person	Virtual	Virtual	Virtual	Virtual	Virtual	In-person
Location	Social Setting (e.g., Faculty Club)	Online (MS Teams)	Online (MS Teams)	Online (MS Teams)	Online (MS Teams)	Online (MS Teams)	Social Setting (e.g., Faculty Club)
Length	60min	30min	90min	90min	90min	30min	60min
Purpose	Ice-breaker and informal social opportunity for group to learn more about each other	Defining coaching and setting expectations	Define professional development topic	Explore what is working now	Articulate plan to achieve the “Dream Vision”	How has coaching shown up since the sessions? How could coaching continue to support you? Offer of 1:1 coaching going forward if desired	Informal social opportunity for the group to reconnect and share experiences
Theme/content	Share positive story, listen to peer’s stories, connect as a group	Explore topic for group sessions, Complete coaching agreement Completion of pre-course evaluation	Clarify what is important about professional development topic and what successful group coaching on this topic looks like – explore this theme with group	Explore the “Dream Vision” for this topic – what works now? What does an ideal vision of this topic look like for you?	Develop accountability structure to ensure next steps will be pursued – goals should be specific, measurable, achievable, relevant, and time-bound	What next? Was there movement towards the envisioned outcome? Completion of post-course evaluation	Overall Feedback Should the group continue to meet?
Participant examples			Topic: Build effective teams and collaborative relationships	“Dream Vision”: Create psychologically safe space in which a diverse team works towards a shared vision	Next Steps: create a list of three achievable tasks to move toward the goal each evening, and review progress from the day before	Using coaching approach in interactions with staff and mentees Desire for individual coaching to help with plan moving forward	Cohort 3 decided to continue to meet independent of the program for accountability and support

Feedback from our focus group and research track faculty in general indicates that early career researchers often feel isolated and without peer support, especially when looking to establish their research independence in a large academic institution [22]. Therefore, group coaching was preferable to 1:1 sessions, since it allowed for the additional benefits of peer support and community building alongside the overall coaching provision. Additionally, group coaching is an economically effective model since it employs one coach for a group, thereby enhancing feasibility and longevity of institutional commitment. Group coaching participants were also offered individual coaching follow-up after participating in the program.

The biggest barrier identified by the focus group was time commitment, since the diverse responsibilities of clinician scientists and researchers often result in an inability to attend extensive sessions or in-person events. Consequently, the program was designed as a modest commitment of three main group coaching sessions with additional shorter meetings around the core components to accommodate clinical and research commitments (Table 1). To honor our goal of meeting researchers “where they are,” the course adopted a blended delivery model, leveraging both in-person and virtual components (Table 1). This blended style of program delivery has been broadly endorsed as effective by faculty to support research learning goals [23]. We received similar feedback from our cohort of participants engaged in the co-creation of this program.

During the initial group coaching cohort, faculty expressed lack of knowledge regarding the purpose of coaching and how it differed from mentoring. Therefore, in the second iteration, we introduced a 30-minute, 1:1 pre-course session to set expectations for the coach and participant, as well as to allow the participants to clarify program objectives in relation to their own goals (Figure 1, Table 1) in subsequent cohorts. A coaching agreement was signed during this meeting to codify expectations and provide accountability for both parties (Supplemental Material 1). In addition to the 1:1 pre-course session, a 1:1 post-course session was provided to offer closure for the coaching intervention. This post-course session focused on personal accountability and determining how coaching could serve the participant after the course was completed (Table 1).

The core group coaching course consisted of three 90-minute sessions which are designed around the appreciative inquiry framework of Define, Discover, Dream, Design, and Destiny/Delivery [19,20]. During these three sessions, participants identified their professional development topic, explored what is/is not currently working, imagined the “dream vision” for this topic, articulated a plan for achieving this vision, and implemented the plan [24] (Table 1). Using the appreciative inquiry framework and guidance from the Office of Faculty Wellness, we encouraged participants to frame their future plans in a positive light, rather than focusing on past failures or negativity.

Feedback from Cohort 2 indicated that introducing an in-person meeting would foster relationships and group connection. This suggestion was implemented for Cohort 3, with an initial in-person pre-course meeting. Subsequent 1:1 and core coaching sessions were conducted virtually to help accommodate clinical and research schedules. The final post-course session again brought the group together in-person (Figure 1, Table 1). The final group session was in person three months after the final core group coaching session (Table 1). This informal social meeting was designed to give the group an opportunity to reconnect, provide feedback on events since course completion, and allow discussion and accountability for achieving their future goals.

### Program participants and group size

In Spring 2023, a call was put out for interested applicants to the group coaching program. Initial efforts focused on early career clinician scientists and laboratory-based researchers as part of an intensive career development program at UTSW for this population. Communications were promoted using established clinical research channels, institutional websites, and existing research training programs. The clinical scholar and research track community was targeted since this group encompasses the majority of faculty with protected time for research at UTSW. Application for group coaching was specifically designed as a self-selection process to support the commitment of participants, promote retention, and active engagement of the group. Group sizes were limited to no more than nine participants at similar career stages, focusing primarily on early career faculty (i.e., instructors and assistant professors).

### Coach qualifications and follow-up

Coaching sessions were led by two certified professional coaches (CPCs) who are established academic faculty that have completed the UTSW Coach Certificate Program (UCCP) through the UTSW Office of Faculty Wellness, a level-1 education International Coaching Federation (ICF) accredited program [25,26]. Following completion of the group coaching course, participants are offered the opportunity to continue their development with 1:1 coaching provided through the UTSW Office of Faculty Wellness where a pool of CPCs are available for ongoing faculty support.

### Program evaluation

To evaluate the group coaching program, we utilized a dual-pronged approach, collecting quantitative and qualitative data to assess feasibility and satisfaction, impact on participant burnout, and ongoing quality improvement. Evaluations were sent to all program participants before and after the coaching intervention using the Research Electronic Data Capture system (REDCap) [27]. A personalized link was sent to all participants, so measures before and after the intervention could be linked to each individual. Basic demographic information was collected with the initial survey. Feasibility was assessed through measuring course interest, enrollment, and completion. Quantitative measures included validated assessment tools (e.g., Professional Fulfillment Index [PFI]) [28], which were utilized to assess professional fulfillment, wellness, and burnout. Additionally, post-course questionnaires covered questions about satisfaction, coach effectiveness, and likeliness to recommend the course to a peer or colleague on a 5-point Likert Scale. Qualitative feedback was gathered post-course via free text questions around the impact of group coaching, what was most or least helpful, and feedback on course format (Supplemental Material 2). To encourage completion of evaluation measures, we provided time in the 1:1 pre-course and post-course sessions for survey completion (Table 1). The number of participants who have completed the course to date does not allow for accurate analysis of burnout or professional fulfillment, so no data on these outcomes will be reported in this manuscript. For the purposes of this report, we focused on feasibility, effectiveness of coaching, and satisfaction, alongside qualitative feedback.

### Institutional commitment

Through the Office of Faculty Wellness, UTSW had a strong foundation of coaching support and excellence. Therefore, support was already established at an institutional level for faculty coaching interventions including free 1:1 coaching offered for all faculty, training in coaching skills, and integrated coaching into faculty development programs. Institutional support, combined with communications and championship from leadership, underlined the importance of coaching for early career faculty. Furthermore, we partnered with existing UTSW career development programs to integrate the group coaching program. This integration incorporated group coaching into institutional career development initiatives for early career investigators, including our clinical research education programs for early career faculty; the NIH Clinical Translational Science Award and the KL2 Scholars Program; and institutional scholarly programs, such as the Dean’s Scholars in Clinical Research and our early R01 investigator program. Integration was achieved through the inclusion of Group Coaching directly in the scholar personalized development plans associated with the above programs.

## Results

### Group coaching program uptake

The UTSW group coaching program was launched in Summer 2023 and has completed three cohorts to date in Fall 2023, Spring 2024, and Summer 2024. A total of 55 individuals responded to campus communications with interest in participating in a coaching cohort; of these, 40% (*n* = 22) formally enrolled in one of three courses in a self-selecting manner (Table 2). Of those enrolled, 95% (*n* = 21) went on to complete the full course (Table 2). The most common reason for not signing up was inability to align schedules, due to clinical/research obligations.

**Table 2.** Characteristics and details of group coaching participants

Characteristics	# Number	%
**Group coaching course interest (*n* = 55)**		
Initial interest	55	–
# Signed up	23	42%
# Enrolled in course	22	40%
**Group coaching participant demographics (*n* = 21)**		
# Completed course*	21	95%
**Gender (*n* = 21)**		
Male	8	38%
Female	13	62%
**Job title (*n* = 21)**		
Assistant professor	16	76%
Trainee	3	14%
Professor	1	5%
Other	1	5%
**Academic track (*n* = 21)**		
Clinical scholar	8	38%
Research/tenure	6	29%
Clinician educator	3	14%
N/A	4	19%
**Academic credentials *(n* = 21)**		
MD	12	57%
PhD	4	19%
MD, MPH	3	14%
MD, PhD	1	5%
N/A	1	5%
**Ethnicity (*n* = 21)**		
White	8	38%
Asian	6	29%
Black/African American	1	5%
Not specified	6	29%
**Grant funds (*n* = 21)**		
NIH R01 or similar	4	19%
NIH CDA or similar	5	24%
Foundation/institutional CDA or similar	4	19%

* One participant dropped out due to personal reasons. Values may not add to 100% due to rounding. Abbreviations: CDA = Career Development Award; NIH = National Institutes of Health.

### Group coaching participant demographics

Twenty-one individuals completed the full group coaching intervention between Fall 2023 and Summer 2024 (95%); 62% identified as female, 38% as White, 29% as Asian, and 5% as Black (Table 2). Most participants had an MD (57%) or PhD (19%) and were faculty (assistant professor, 76%; professor, 5%) spread across three UTSW academic tracks. The clinical scholar track (38% of participants) includes clinical faculty who also participate heavily in research (∼50% + of research time), the research/tenure track (29% of participants) includes faculty who predominantly work in research with minimal or no clinical duties (including MD/PhD and PhD researchers with >80% research time), and the clinician educator track (14% of participants) included faculty with a predominant clinical workload and minimal (20% or less) research involvement (Table 2).

### Evaluation completion

Enrollees in the group coaching program were sent evaluations pre- and post-coaching to assess change in burnout and fulfillment (data not reported). Post-intervention questionnaires also evaluated satisfaction and effectiveness of the group coach. To improve response rates, we incorporated these questionnaires into the 1:1 pre- and post-course sessions, which resulted in higher rates of measure completion in the Spring 2024 (6/7 completed, 86%) and Summer 2024 (5/6 completed, 83%) cohorts, as opposed to our first Fall 2023 (5/8 completed, 63%) cohort where this strategy was not implemented. The overall completion rate was 76% (16/21 individuals).

### Coaching feasibility and satisfaction

**Figure 2.**
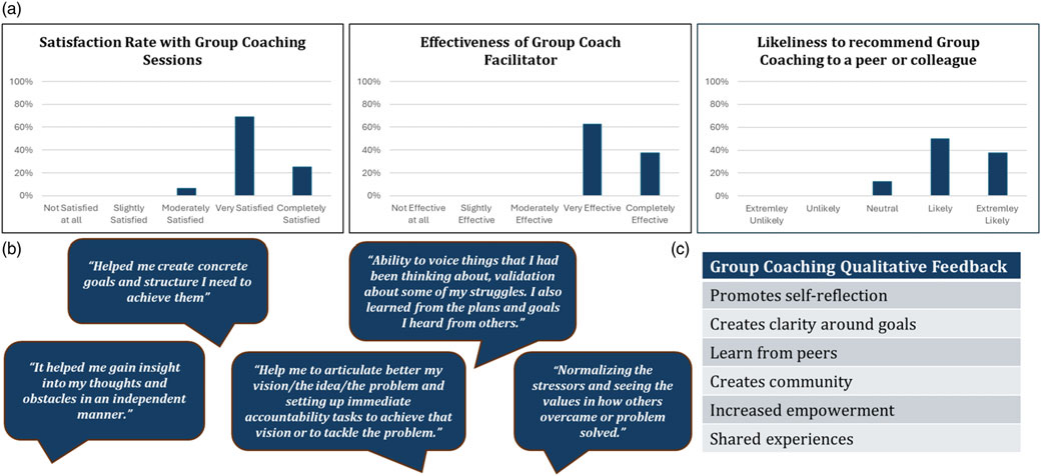
Participant feedback. This figure displays: (***a***) the satisfaction and effectiveness of our group coaching intervention: 16/21 participants completed questionnaires after the conclusion of group coaching and reported high rates of satisfaction and efficacy of their coach; (***b***) qualitative feedback: quotes from participants; and (***c***) qualitative feedback: participants reported favorable experiences centered on the themes of belonging, validation of shared struggles, learning from others, and insight into framing goals, problems, and solutions in a constructive manner.

With high levels of interest, enrollment, and course completion (Table 2), we were able to demonstrate feasibility and appetite for this intervention. Participants indicated that they were either very or completely satisfied with the group coaching sessions (94%) (Figure 2a), with 100% indicating their group coach facilitator was either very or completely effective (Figure 2a). Participants gave their overall experience with group coaching an average rating of 4.5/5, and the majority of individuals shared they were likely to recommend group coaching to a peer or colleague (88%) (Figure 2a).

Participants were asked to provide qualitative feedback about their experience and responses were broadly positive (Figure 2b). A review of the qualitative responses identified several common elements including promotion of self-reflection, clarity around goals, learning from peers and creation of community, increased empowerment, and the importance of shared experiences (Figure 2c). Interestingly, several participants commented they would appreciate the option of an in-person group coaching course (e.g., *“If scheduling allows, I think it would be best for it to be in-person”*) and indicated their appreciation of the organic, personal parts of the course, *“I liked the one-on-ones at the beginning and end, and the introductory in-person session.”*

## Discussion

Results indicate that our group coaching program is feasible and acceptable with significant interest in participation, high completion rates, and high levels of satisfaction. Participants reported effective coaching and endorsed positive feedback themes including enhanced self-reflection, sense of community, shared experience, and increased empowerment, with no detraction from clinical or scientific productivity.

A novel aspect of our group coaching program design was the concept of co-creation using the appreciative inquiry framework (Figure 1, Table 1). To date, coaching programs have been predominantly designed with minimal feedback from their participants [14–16] and have not incorporated the appreciative inquiry framework, which focuses on a positive and hopeful approach to challenges [24]. By “meeting our faculty where they are” and pivoting in response to feedback from each cohort (Figure 1), we were able to create a program that fits the demands of both clinical and research schedules, while simultaneously providing a positive experience for participants (Figure 2a–c). Conducting a hybrid program allowed for flexibility through virtual sessions, while also fostering connection via targeted in-person meetings (Table 1). Interestingly, as the cohorts progressed, participants were requesting more in-person meetings with several pieces of feedback indicating that these meetings *“should be in person.”* This request for connection embodies the spirit of coaching, and in response to this feedback, we have created more in-person sessions (Figure 1, Table 1). We will likely continue to expand these offerings in our next cohort, where we are planning for one of the core 90-minute coaching sessions to take place in-person.

One challenge evaluating this program was determining the most effective set of measures that accurately reflect the attributes modifiable by coaching and relevant to academic faculty. Given our sample size, we have only been able to evaluate satisfaction and effectiveness of coaching to date, although we have collected data on Professional Fulfillment and Burnout as a measure of physician wellness [28–30], which we plan to report when we have an appropriate sample size. Nevertheless, standardized professional fulfillment and burnout measures are not traditionally designed to encompass some of the more positive themes fostered in group coaching such as self-reflection, empowerment, community building, and shared experience [24,28] (Figure 2c). Therefore, measures that effectively capture these less-appraised aspects of coaching are needed. We are currently working with our wider UTSW coaching community to pilot some alternative evaluations to determine if we can more accurately capture the coaching experience.

The group nature of this program provides not only a cost-effective model in utilizing a single coach for multiple individuals, but creates an atmosphere of peer support, bringing diverse faculty together at similar points in their career. This observation agrees with qualitative data on group coaching in the literature [18]. An additional benefit of our group coaching program was creating affinity groups. From each cohort, several participants agreed to meet up outside of their coaching cohorts, with one creating a writing accountability group. This was an organic result of the program, since coaching lends itself to creating supportive peer groups, as described above [18]. Given our coaching cohorts’ successful accountability, we plan to provide an option for a structured meeting if cohorts express the desire to build on their relationships in the future. Such developments demonstrate how this program can serve as a translational research interface for participants.

The group coaching program had greater than expected uptake by researchers across career tracks. Although marketed to clinical scholars, basic science and public health researchers represented a substantial number of participants. We were surprised to see that the intervention proved satisfactory for a wide array of research faculty, with all participants broadly endorsing positive themes and experiences as a result of the intervention (Figure 2a–c).

In conclusion, we have developed a unique, hybrid group coaching program for clinician researchers and scientific faculty that is feasible and highly satisfactory to participants. The UTSW group coaching program continues to be a vital part of research scholar support through integration with institutional research programs and ongoing faculty offerings. The program proved effective for researchers with a broad array of research interests, brought together diverse faculty through their shared experiences, and fostered connections and collaborations within this translational group. Through co-creation with our faculty, we have designed a group coaching program that can be deployed across academic medical centers in an inclusive fashion to help support more fulfilled and empowered researchers. Looking to the future, improved targeted coaching measures are needed.

## Supporting information

10.1017/cts.2025.10089.sm001 Palmer et al. supplementary material 1 Palmer et al. supplementary material

10.1017/cts.2025.10089.sm002 Palmer et al. supplementary material 2 Palmer et al. supplementary material

## Supplementary material

The supplementary material for this article can be found at https://doi.org/10.1017/cts.2025.10089.
