# Supplementary material for: Group coaching for career development: Supporting the endangered early career researcher
Source: J Clin Transl Sci. 2025 Jul 10;9(1):e166. doi: 10.1017/cts.2025.10089 (PMC12392355; doi:10.1017/cts.2025.10089)
Supplement: Palmer et al. supplementary material 1 — Palmer et al. supplementary material [file S2059866125100897sup001.pdf]

Clinical Researcher Academy: Group Coaching Agreement

Coach Facilitator:

Coach: each participant when providing peer coaching

Client: each participant when receiving peer coaching

What is coaching?

Coaching is a transformative process that engages clients in an accountable partnership. This partnership facilitates growth and fulfillment in their professional and personal lives through the identification of who they want to be, the ownership of challenges, and the creation of meaningful action plans for success. A coaching session is designed to ask questions that create space for self-reflection that will turn into action to help you achieve your goals in both your professional and personal life.

What does group coaching look like?

In group coaching, the coach facilitator facilitates the group engaging in peer coaching each other in a real and authentic manner. Each client/participant gives and receives peer coaching to other client/participants in each session. A coaching session is designed to ask questions that create space for self-reflection that will turn into action to help you achieve your goals in both your professional and personal life. The group participants receive the benefits of being coached as well as acquire skills on how to bring a coaching culture to their environments such as their research and/or clinical teams, peer groups, trainees, direct reports, etc. The group determines the coaching topics most relevant to them, within the context of professional development as a faculty member. The UTSW Clinical Research Accelerator is providing this service to facilitate professional development.

Examples of coaching topics:

- Accountability
- Commitment
- Communicating
- Focus
- Goal setting and prioritization
- Handling Conflict
- Leadership
- Negotiation
- Organization

- Self Confidence
- Self-Efficacy
- Social Engagement
- Time Management
- Work-Life Balance

What is the time commitment?

Group coaching will occur over 5-6 sessions at a frequency determined by the coach facilitator and the group. One on one meetings will occur between each client and the coach facilitator once prior to the group sessions and once after completion of the group sessions. Typical group sessions last 1 hour and occur weekly or biweekly. Individual 1:1 coaching can also be arranged separate from these group coaching sessions.

What are my responsibilities?

To come to the sessions ready to work with your peers and to be open to discovering your best self.

Is information shared in coaching sessions confidential?

All coaching session information will be kept confidential and will not be shared with anyone. If there are concerns for your safety or the safety of others or if you share information about sexual misconduct that requires reporting under Title IX, these will be reported as required by university policy.

Group Coaching Partnership Agreement

1. **Scope:** As Coach and Client, we agree to meet as a group for one hour per session for 6 sessions over the time period determined by the Clinical Research Accelerator, the coach, and the group. Each Client will meet briefly for 15-30 minutes 1:1 with the Coach once prior to the group sessions and once after completion of the sessions. All group coaching sessions are conducted virtually. To ensure the objectives of group coaching are met for all participants, no group session can be missed except for extenuating circumstances. At the end of the engagement, the Client may request 1:1 coaching sessions, subject to availability. Please keep your coach informed of your intentions.

2. **Presence:** To ensure the objectives of group coaching are met, in particular that each Client receives individual peer coaching to facilitate their own growth, awareness, and insight, each Client must be fully present, engaged, and authentic in each session. Given the virtual format, this includes the following:

> logging in 5 minutes prior to each session to allow the full hour to be used for coaching. Attending through the end of each session.

> enabling the video during the entire session. During some aspects of the sessions, instructions to mute video/audio will be given.

> ensuring there are no competing responsibilities or distractions during the sessions. This includes being free of active clinical duties, including responding to pagers, being in a space without intrusion from others, including family members and pets, and not responding to texts or calls or engaging in general with personal devices.

> Clients will coach each other during each session. Clients are expected to actively engage both as recipients of coaching and as peer coaches during each session. The topics will be determined by the group. The nature of each coaching session should be authentic and not role-play.

2. **Scheduling**: Scheduling of all coaching sessions will be coordinated by the UTSW Clinical Research Accelerator staff. It is the responsibility of the Client to ensure the sessions are on their calendar with the virtual link with adequate reminders.

3. **Cost**: Professional Group Coaching is provided at no cost to the Client or client's department. Funding for Professional Group Coaching is provided by the UTSW Clinical Research Accelerator.

4. **Confidentiality:** All coaching session information will be kept confidential and will not be shared with anyone by the Coach. If there are concerns for your safety or the safety of others or if you share information about sexual misconduct that requires reporting under Title IX, these

will be reported as required by university policy. Clients will not be allowed to share content of the coaching sessions with anyone outside the group coaching participants.

5. **Nature of Coaching**: Coaching consists of focused dialogue between Peer Coach and Client, designed to develop self-insight and purposeful action toward the Client's goals. The Peer Coach will work with the Client to define their goals and hold them accountable for commitments made, but Client is ultimately responsible for their own learning and development. Coaching is not training, advising, mentoring, or knowledge transfer, though those outcomes can coincide with the coaching dialogue. During Peer Coaching, giving direct advice or solutions should be avoided.

6. **Misunderstandings:** As a Coach, we are human - limited by our own background, identities, and experiences – so we will at times misunderstand the Client's experience. As the Coach Facilitator, I will be checking in with you about this and I ask that you tell me when I miss the mark or cause offense.

While these expectations cover the basics, there will continue to be topics and issues which require clarification and further agreement. The Coach and Client agree to openly raise -- and transparently discuss -- any concerns or issues which arise during the coaching engagement.

______________________________ ______________________________

Coaching Client Coach Facilitator
