# Supplementary material for: Group coaching for career development: Supporting the endangered early career researcher
Source: J Clin Transl Sci. 2025 Jul 10;9(1):e166. doi: 10.1017/cts.2025.10089 (PMC12392355; doi:10.1017/cts.2025.10089)
Supplement: Palmer et al. supplementary material 2 — Palmer et al. supplementary material [file S2059866125100897sup002.pdf]

Clinical Researcher Academy Coaching Survey

To enable us to help gauge the impact of group coaching and follow faculty through the Clinical Researcher Academy experience, please complete the following survey questions which are validated instruments regarding wellness, burnout, and professional development.

Identifiable data from this survey will remain within the Clinical Researcher Academy and will not be shared with your Mentoring Team, Department or outside of the Academy. Any data used for review or publication would be fully anonymized.

First Name ______________________________

Last Name ______________________________

Please provide the email to which you would like your survey to be sent ______________________________

Which best represents your role at UTSW?
- ◯ Faculty
- ◯ Resident
- ◯ Fellow
- ◯ Staff

Overall, do you consider yourself to work: ◯ Full-time ◯ Part-time

How true do you feel the following statements are about you at work during the past two weeks?

	Not at all true	Somewhat true	Moderately true	Very true	Completely true
I feel happy at work	○	○	○	○	○
I feel worthwhile at work	○	○	○	○	○
My work is satisfying to me	○	○	○	○	○
I feel in control when dealing with difficult problems at work	○	○	○	○	○
My work is meaningful to me	○	○	○	○	○
I'm contributing professionally (e.g., patient care, teaching, research, and leadership) in the ways I value most	○	○	○	○	○

PFI Score (mean([pfi_1],[pfi_2],[pfi_3],[pfi_4],[pfi_5],[pfi_6]) *2.5) ________________________________

To what degree have you experienced the following?

During the past two weeks, I have felt...

	Not at all	Very little	Moderately	A lot	Extremely
A sense of dread when I think about work I have to do	○	○	○	○	○
Physically exhausted at work	○	○	○	○	○
Lacking in enthusiasm at work	○	○	○	○	○
Emotionally exhausted at work	○	○	○	○	○

Burnout Score Emotional Exhaustion
mean([bo_ee_1],[bo_ee_2],[bo_ee_3],[bo_ee_4])*2.5 ________________________________

During the past two weeks, my job has contributed to me feeling...

	Not at all	Very little	Moderately	A lot	Extremely
Less empathetic with my patients	○	○	○	○	○
Less empathetic with my colleagues	○	○	○	○	○
Less sensitive to others' feelings/emotions	○	○	○	○	○
Less interested in talking with my patients	○	○	○	○	○
Less connected with my patients	○	○	○	○	○
Less connected with my colleagues	○	○	○	○	○

Burnout interpersonal disengagement ________________________________

Individual Burnout Score ________________________________

Survey Continued

	Highly Unlikely	Unlikely	Neither Likely nor Unlikely	Likely	Highly Likely
What is the likelihood that you will leave UTSW within the next two years?	○	○	○	○	○

**Please answer the following questions based on your experience at work over the past month.**					
	Strongly Disagree	Disagree	Neither Agree or Disagree	Agree	Strongly Agree
I have felt unheard, invisible, or un-noticed	◯	◯	◯	◯	◯
I am able to speak in meetings without undue interruptions	◯	◯	◯	◯	◯
I have had a voice in matters that are important to me	◯	◯	◯	◯	◯
In my organization, I feel like I matter	◯	◯	◯	◯	◯
I feel that I belong here	◯	◯	◯	◯	◯
I have been well mentored	◯	◯	◯	◯	◯

How has your job affected your personal relationships during the past month?

In the past month, my job has...

	Not at all true	Somewhat true	Moderately true	Very true	Completely true
Made it harder for me to nurture existing personal relationships	○	○	○	○	○
Made it harder for me to develop new meaningful personal relationships	○	○	○	○	○
Contributed to conflict in my personal relationship(s)	○	○	○	○	○
Contributed to me feeling more isolated or detached from the people who are most important to me	○	○	○	○	○

How true are the following statements about conditions in your practice setting (i.e., your principal practice site)?

	Not all true	Somewhat true	Moderately true	Very true	Completely true
My input is valued in important administrative decisions	○	○	○	○	○
Our organizational goals and values fit well with my goals and values	○	○	○	○	○
Administration values my clinical work	○	○	○	○	○

Group coaching feedback:

How satisfied were you with the group coaching sessions?

◯ Not satisfied at all ◯ Slightly satisfied ◯ Moderately satisfied ◯ Very satisfied ◯ Completely satisfied

How has group coaching impacted your life thus far?

What was the most helpful about group coaching?

What was the least helpful about group coaching?

What would you recommend to improve the format of group coaching?

How likely are you to recommend group coaching to a peer/colleague?

◯ Extremely Unlikely ◯ Unlikely ◯ Neutral ◯ Likely ◯ Extremely Likely

How likely are you to pursue 1:1 coaching?

◯ Extremely Unlikely ◯ Unlikely ◯ Neutral ◯ Likely ◯ Extremely Likely

Group Coach Facilitator feedback:

How effective was the group coach?

◯ Not effective at all ◯ Slightly effective ◯ Moderately effective ◯ Very effective
◯ Completely effective

What did you appreciate most about your group coach?

In what way could your group coach improve?

On a scale of 1 to 5, how would you rate your overall experience with this coaching program?

poor neutral excellent

(Place a mark on the scale above)

Is there anything that you would like for us to know about your experience of group coaching?
